# Sources of Dietary Protein in Relation to Blood Pressure in a General Dutch Population

**DOI:** 10.1371/journal.pone.0030582

**Published:** 2012-02-07

**Authors:** Wieke Altorf - van der Kuil, Mariëlle F. Engberink, Moniek M. Vedder, Jolanda M. A. Boer, W. M. Monique Verschuren, Johanna M. Geleijnse

**Affiliations:** 1 Top Institute Food and Nutrition, Wageningen, The Netherlands; 2 Division of Human Nutrition, Wageningen University, Wageningen, The Netherlands; 3 National Institute for Public Health and the Environment (RIVM), Bilthoven, The Netherlands; Universidad Peruana Cayetano Heredia, Peru

## Abstract

**Background:**

Little is known about the relation of different dietary protein types with blood pressure (BP). We examined whether intake of total, plant, animal, dairy, meat, and grain protein was related to BP in a cross sectional cohort of 20,820 Dutch adults, aged 20–65 y and not using antihypertensive medication.

**Design:**

Mean BP levels were calculated in quintiles of energy-adjusted protein with adjustment for age, sex, BMI, education, smoking, and intake of energy, alcohol, and other nutrients including protein from other sources. In addition, mean BP difference after substitution of 3 en% carbohydrates or MUFA with protein was calculated.

**Results:**

Total protein and animal protein were not associated with BP (p_trend_ = 0.62 and 0.71 respectively), both at the expense of carbohydrates and MUFA. Systolic BP was 1.8 mmHg lower (p_trend_<0.01) in the highest (>36 g/d) than in the lowest (<27 g/d) quintile of plant protein. This inverse association was present both at the expense of carbohydrates and MUFA and more pronounced in individuals with untreated hypertension (−3.6 mmHg) than in those with normal (+0.1 mmHg) or prehypertensive BP (−0.3 mmHg; p_interaction_<0.01). Meat and grain protein were not related to BP. Dairy protein was directly associated with systolic BP (+1.6 mmHg, p_trend_<0.01), but not with diastolic BP (p_trend_ = 0.24).

**Conclusions:**

Total protein and animal protein were not associated with BP in this general untreated Dutch population. Plant protein may be beneficial to BP, especially in people with elevated BP. However, because high intake of plant protein may be a marker of a healthy diet and lifestyle in general, confirmation from randomized controlled trials is warranted.

## Introduction

Elevated blood pressure (BP) is a major risk factor for cardiovascular disease and renal impairment. It has been estimated that already from systolic BP levels as low as 115 mmHg onward, risk of cardiovascular disease increases linearly with increasing BP [1]. Therefore, health authorities emphasize the importance of dietary and lifestyle interventions beneficially influencing BP including physical activity, obtaining a healthy body weight, moderate alcohol consumption, reduced salt intake, and increased potassium intake [2], [3]. More recently, interest has grown into dietary patterns and macronutrient intakes, including dietary protein [4], [5]. A substantial body of evidence suggests a, possibly weak, beneficial effect of protein on BP, although findings are not conclusive [6], [7].

Protein intake is a rather heterogeneous exposure and types of protein (i.e. animal and plant protein and protein from specific sources like dairy, meat, grain) might differentially influence BP. In several observational studies [8], [9], [10], [11], [12], [13], [14] the association with BP was investigated separately for plant protein and animal protein. Results were inconclusive, although there is a trend towards a slightly more beneficial effect of plant protein than of animal protein on BP. Data on specific protein sources in relation to BP are scarce. We observed no association between intake of dairy, meat, and grain protein with 6-year incidence of hypertension in a previous analysis including 2241 adults (≥55 y) from the population based Rotterdam Study [12]. He et al. recently published findings of a randomized, double-blind cross-over trial among 352 prehypertensive and hypertensive participants in which BP effects of supplementation with soy protein, milk protein and complex carbohydrates was investigated [15]. Compared with carbohydrate, soy protein and milk protein (40 g/d) resulted in a −2.0 mmHg and −2.3 mmHg net change in systolic BP, respectively, but the achieved BP reductions did not differ between soy and milk protein supplementation.

BP response to protein intake may differ between population subgroups, which may be an important issue because of public health recommendations [5], [16]. In the INTERSALT study among 10,020 adults from 32 countries the inverse association between protein intake and BP was more pronounced in participants aged >40 y than in younger participants [16]. Furthermore, in the OmniHeart trial in 164 adults, BP reductions during a high-protein diet were larger in hypertensive participants than in prehypertensive participants [5]. However, more research is needed to be able to draw firm conclusions on potentially sensitive population subgroups.

In the present analysis, we examined whether intake of total protein, plant protein, animal protein, and protein from specific sources was related to BP level in a general Dutch population of 20,820 adults. With respect to protein sources our main focus was on protein from dairy, meat, and grain, as these are the main sources of animal and plant protein in the Netherlands [17]. Additionally, we assessed whether protein-BP associations were modified by gender, age, BMI, and BP level.

## Methods

### Study population

We used data from the population-based Monitoring Project on Risk Factors for Chronic Diseases (MORGEN project), which is part of the Dutch EPIC cohort. Details of the study have been described elsewhere [18]. In brief, between 1993 and 1997 22,606 men and women aged 20–65 y completed questionnaires on diet, lifestyle, and health and underwent a physical examination. The Medical Ethics Committee of the Netherlands Organization for Applied Scientific Research (TNO) approved the study protocol and all participants signed informed consent form. We excluded 16 participants with missing data on BP and 1,093 participants who used antihypertensive medication. Additionally, we excluded 677 participants who were diabetic, had a history of myocardial infarction or stroke, or were pregnant, leaving 20,820 men and women for the present analyses.

### Dietary assessment and exposure categories

Dietary intake was assessed using a self-administered semi-quantitative food frequency questionnaire (FFQ) on 178 foods and beverages consumed during the preceding year [19]. Colored photographs were used to facilitate estimation of portion sizes, and seasonal variation in food intake was taken into account. Total energy and nutrient intakes were calculated using an extended version of the Dutch Food Composition Table of 1996 [20].

Animal protein was defined as protein from dairy, meat, fish, eggs, and animal protein from mixed dishes. Plant protein included protein from grain, potatoes, fruits, vegetables, nuts, legumes, soy, and plant protein from mixed dishes. Dairy protein was calculated as protein from all kind of milk, yogurt, coffee creamer, curd, pudding, porridge, custard, ice-cream, whipped cream, and cheese. Meat protein included protein from all meat and meat based products, and grain protein was calculated as plant protein from rice, bread, pasta and bakery products. In addition, we calculated protein from potatoes (including fries), vegetables, fruits, and legumes (without green beans and peas).

In a validation study among 63 men and 58 women good reproducibility was shown for energy adjusted total protein intake with Pearson correlation coefficients of 0.73 in men and 0.70 in women [21]. The relative validity of the FFQ was assessed against 12 monthly 24-h recalls over a 1-year period. Pearson correlation coefficients for energy adjusted total protein intake after correction for intra-individual variation were 0.71 for men and 0.67 for women [21]. Energy adjusted total protein intake as assessed from the FFQ also correlated well with urinary nitrogen excretion in four 24 h urine samples at 3-month intervals, i.e. Pearson correlation coefficients of 0.56 for men and 0.69 for women [21]. For types of protein the FFQ was not validated, but correlations for milk and milk products and bread, as surrogate markers for dairy and grain protein, were good (all r>0.65), whereas correlations for meat were lower, especially for men (r_men_ = 0.39; r_women_ = 0.59) [19].

### Blood pressure

Systolic and diastolic BP (first and fifth Korotkoff sounds, respectively) was measured by trained nurses using a random zero sphygmomanometer on the left arm in supine position, after a 5-minute rest. BP was measured twice, 30 seconds apart, and the mean of the two readings was used. During physical examination, regular audits were performed to check adherence to the BP measuring protocol (e.g. resting time, adequate cuff size). Normotension was defined as systolic BP≤120 mmHg and diastolic BP≤80 mmHg. Hypertension was defined as a systolic BP of ≥140 mmHg and/or diastolic BP of ≥90 mmHg (participants using antihypertensive medication were excluded). All other participants were considered to be prehypertensive.

### Lifestyle factors

Body weight (to nearest 0.1 kg) and height (to nearest 0.5 cm) were measured with participants wearing light indoor clothing without shoes and body mass index (BMI) was calculated (kg/m^2^). Data on age, gender, education, lifestyle factors, history of major diseases, medication use, and any prescribed diets were collected by questionnaires. A questionnaire on physical activity pattern in the preceding year was introduced in 1994 and was completed by 16,073 participants (77%) of our cohort. Participants were classified in categories of alcohol intake (none, moderate, high), smoking status (current smoker/non-smoker), educational level (3 categories), and physical activity (4 categories, ranging from inactive to very active [18]).

### Statistical analysis

Data analysis was performed using SAS version 9.1 (SAS Institute Inc.). Protein intake was first adjusted for total energy intake according to the residual method [22]. Baseline characteristics of the study population were calculated in quintiles of energy-adjusted total protein intake, and are presented as means ± standard deviation, percentages, or medians with interquartile range.

We used general linear models to calculate average BP levels with 95% confidence intervals (CI) in quintiles of energy-adjusted protein intake (total, animal, plant, dairy, meat and grain). The basic model (model 1) included age and gender. In model 2, further adjustment was made for BMI, education, smoking, and alcohol consumption. The fully adjusted model (model 3) additionally included daily intake of total energy, saturated fatty acids, carbohydrates, fiber, calcium, magnesium, potassium, and protein intake from other sources than the one under study. Because grain comprised only 48% of plant protein intake we conducted post hoc analyses in which we calculated fully adjusted mean BP in tertiles of dietary protein intake from potatoes, vegetables, fruits, and legumes.

To investigate whether physical activity confounded the protein-BP associations, post hoc analyses were conducted per 5 grams of total, animal and plant protein in the subgroup with data on physical activity using the full model (model 3) with or without additional adjustment for physical activity. In addition we performed substitution analyses to investigate the BP difference with exchange of nutrients. By including total protein and carbohydrate as continuous variables in the same multivariable model (model 3) we investigated the BP difference with 3 energypercentage (en%) higher total protein intake at the expense of carbohydrates. The difference in the coefficients of total protein and carbohydrates plus their covariance was used to estimate BP difference and 95% confidence interval for the substitution. Similarly we investigated the BP difference of 3 en% higher total protein at the expense of mono-unsaturated fat. The same substitution analyses were performed for animal protein and plant protein.

Finally, for total, plant and animal protein, pre-defined subgroup analyses were performed in strata of gender, age (<50 y and ≥50 y), BMI (<25 kg/m^2^ and ≥25 kg/m^2^), and BP level (normotensives, prehypertensives and untreated hypertensives), using the full model (model 3).

## Results

### Descriptive statistics

The mean age of the population was 42±11 y and 45% were men. Average BP was 120.0±15.6 mmHg systolic and 76.1±10.4 mmHg diastolic, and 15% of the population had untreated hypertension. The mean energy-adjusted total protein intake of the study population was 84±12 g/d (∼15 energy%), with 52±13 g/d derived from animal sources. After energy adjustment of dietary protein, age and sex adjusted Pearson partial correlation coefficients were 0.89 for total protein with animal protein, 0.07 for total with plant protein, and −0.39 for plant protein with animal protein. Major sources of animal protein intake were dairy (42%) and meat (40%). Plant protein intake mainly comprised grain protein (48%), whereas other sources were potatoes (10%), vegetables (7%), fruits (4%), and legumes (2%).

Participants with a higher intake of total protein had a somewhat higher BP and were more likely to be overweight or obese, whereas they were less likely to be a current smoker than participants with a low intake (**Table 1**). Fat intake and carbohydrate intake did not differ between quintiles, whereas higher intake of protein was accompanied with higher intake of minerals (i.e. calcium, magnesium, and potassium).

**Table 1:** Characteristics by quintiles of energy adjusted total protein intake of 20,820 Dutch adults.

	Quintiles of energy adjusted total protein intake (g/d)				
	<74 (n = 4173)	74 to 81 (n = 4166)	81 to 86 (n = 4159)	86–93 (n = 4166)	>93 (n = 4156)
Median intake, g/d	70	78	83	89	98
Age, y	41±11	42±11	42±11	43±11	43±11
Gender, % male	49	43	42	43	50
High education, %	22	26	26	26	22
Systolic BP, mmHg	119.5±15.6	119.5±15.8	119.6±15.9	120.8±15.7	120.7±15.3
Diastolic BP, mmHg	75.8±10.3	75.7±10.3	76.0±10.4	76.7±10.5	76.5±10.3
Hypertension, %^1^	13.6	14.1	14.5	15.8	15.6
Body mass index, kg/m^2^	24.0±3.6	24.5±3.8	24.9±3.7	25.2±3.8	25.8±4.1
Overweight, %	35	38	43	48	53
High physical activity, %^2^	9±12	8±11	8±11	9±11	10±13
Alcohol among consumers, glass/d^3^^,^ 4	2.0 (1.0–3.6)	1.4 (0.7–2.9)	1.4 (0.7–2.4)	1.3 (0.7–2.1)	1.3 (0.7–2.1)
Current smoking, %	46	38	36	30	32
Dietary intake					
Total energy, kJ/day	10186±3282	9204±2799	9054±2634	9157±2589	10131±3157
Total protein, g/d (en%)	72±23 (12)	75±21 (14)	80±20 (15)	86±20 (16)	105±27 (18)
Animal protein, g/d (en%)	40±15 (7)	44±14 (8)	49±13 (9)	55±12 (11)	71±19 (12)
Plant protein, g/d (en%)	33±12 (5)	31±10 (6)	31±10 (6)	31±10 (6)	34±12 (6)
Dairy protein, g/d (en%)^5^	14±9 (2)	17±9 (3)	20±10 (4)	24±10 (6)	33±16 (6)
Meat protein, g/d (en%)^6^	16±10 (3)	18±10 (3)	20±9 (4)	22±10 (5)	27±12 (5)
Grain protein, g/d (en%)^7^	15±7 (2)	15±7 (3)	15±6 (3)	15±7 (3)	17±8 (3)
Total fat, g/d (en%)	95±37 (35)	87±31 (36)	87±30 (36)	88±29 (36)	97±35 (36)
Saturated fat, g/d (en%)	38±15 (14)	35±13 (15)	36±13 (15)	36±12 (15)	41±16 (15)
Carbohydrates, g/d (en%)	288±96 (48)	254±80 (47)	245±77 (46)	245±75 (44)	263±92 (44)
Fiber, g/d	24±8	24±7	24±7	25±7	27±8
Calcium, mg/d	849±340	918±342	1014±357	1145±382	1498±581
Magnesium, mg/d	350±110	345±97	354±92	370±94	423±117
Potassium, mg/d	3534±1037	3493±927	3587±867	3762±885	4294±1090

Unless indicated otherwise, data are presented as mean ± SD or %.

1 Hypertension is defined as systolic BP≥140 mmHg or diastolic BP≥90 mmHg (participants using antihypertensive medication were excluded).

2 Data from a subgroup (n = 16,073). In consecutive quintiles n = 3,255, n = 3,229, n = 3,190, n = 3,184, and n = 3,215. High physical activity was defined as ≥3.5 hours moderate activity (4.0>MET≥6.5) and ≥2 h/wk vigorous activity (MET≥6.5).

3 Percentage of alcohol consumers in consecutive quintiles 63%, 63%, 63%, 60% and 58%.

4 Presented as median with interquartile range because of skewed distribution.

5 Protein intake from all kind of milk, yogurt, coffee creamer, curd, pudding, porridge, custard, whipped cream, and cheese.

6 Protein intake from all kind of meats, meat products and poultry.

7 Plant protein intake from all kinds of breads, cake and cookies, grains and grain products.

### Protein intake and blood pressure

Intake of total and animal protein was not clearly associated with BP (**Table 2**), whereas in the highest quintile of dietary plant protein mean BP was −1.8/−1.0 mmHg lower than in the lowest quintile (p_trend_<0.01). Sensitivity analysis within the subgroup of 16,073 participants for whom data on physical activity were available, showed essentially similar estimates when physical activity was additionally included in the multivariable model.Beta's for systolic BP per 5 grams of total protein was 0.13±0.06 mmHg with physical activity in the model versus 0.14±0.05 without physical activity. For animal and plant protein beta's per 5 grams were 0.15±0.03 versus 0.16±0.02 mmHg and −0.43±0.005 versus −0.41±0.006 respectively.

**Table 2:** Fully adjusted systolic and diastolic BP levels in 20,820 untreated Dutch adults in quintiles of energy adjusted total, animal and plant protein intake.

	Median intake(g)	Model 1		Model 2		Model 3	
		SBP	DBP	SBP	DBP	SBP	DBP
**Total protein**							
Q1	69	119.6 (119.2–120.0)	75.9 (75.6–76.2)	120.2 (119.8–120.6)	76.5 (76.2–76.8)	120.1 (119.6–120.6)	76.2 (75.9–76.5)
Q2	78	119.8 (119.4–120.2)	75.9 (75.6–76.2)	120.1 (119.7–120.5)	76.2 (75.9–76.4)	120.2 (119.7–120.6)	76.1 (75.8–76.3)
Q3	83	119.8 (119.4–120.3)	76.1 (75.8–76.4)	119.8 (119.4–120.2)	76.1 (75.8–76.4)	119.9 (119.5–120.3)	76.1 (75.8–76.4)
Q4	89	120.7 (120.2–121.1)	76.6 (76.3–76.9)	120.5 (120.0–120.9)	76.4 (76.1–76.7)	120.5 (120.1–120.9)	76.5 (76.2–76.8)
Q5	98	120.1 (119.7–120.5)	76.2 (75.9–76.5)	119.4 (118.9–119.8)	75.6 (75.3–75.9)	119.3 (118.8–119.8)	75.9 (75.5–76.2)
*P trend*		0.01	0.02	0.03	<0.01	0.14	0.62
**Animal protein**							
Q1	36	119.0 (118.5–119.4)	75.4 (75.1–75.7)	119.8 (119.4–120.3)	76.1 (75.8–76.4)	119.9 (119.4–120.4)	76.0 (75.7–76.4)
Q2	45	119.7 (119.3–120.2)	76.0 (75.8–76.3)	120.0 (119.6–120.5)	76.3 (76.0–76.6)	120.1 (119.7–120.5)	76.2 (75.9–76.5)
Q3	51	120.3 (119.9–120.7)	76.4 (76.1–76.7)	120.3 (119.9–120.7)	76.4 (76.1–76.7)	120.3 (119.9–120.7)	76.3 (76.1–76.6)
Q4	58	120.6 (120.1–121.0)	76.4 (76.1–76.7)	120.4 (119.9–120.8)	76.2 (76.0–76.5)	120.4 (119.9–120.8)	76.3 (76.0–76.5)
Q5	68	120.4 (120.0–120.8)	76.4 (76.1–76.7)	119.4 (119.0–119.9)	75.7 (75.4–76.0)	119.3 (118.8–119.8)	75.9 (75.5–76.2)
*P trend*		<.001	<.001	0.32	0.04	0.39	0.71
**Plant protein**							
Q1	25	120.9 (120.5–121.3)	76.6 (76.3–76.9)	120.6 (120.2–121.0)	76.5 (76.2–76.8)	121.0 (120.4–121.5)	76.6 (76.2–76.9)
Q2	29	120.4 (119.9–120.8)	76.5 (76.2–76.8)	120.0 (119.6–120.4)	76.3 (76.0–76.6)	120.2 (119.8–120.7)	76.3 (76.0−76.6)
Q3	32	120.3 (119.9–120.7)	76.5 (76.2–76.8)	120.1 (119.7–120.6)	76.3 (76.0–76.6)	120.2 (119.8–120.6)	76.3 (76.0–76.6)
Q4	34	119.4 (118.9–119.8)	75.9 (75.6–76.2)	119.6 (119.1–120.0)	75.9 (75.6–76.2)	119.4 (119.0–119.9)	75.9 (75.6–76.2)
Q5	39	119.0 (118.6–119.4)	75.3 (75.0–75.6)	119.6 (119.2–120.1)	75.6 (75.3–75.9)	119.2 (118.6–119.7)	75.6 (75.2–76.0)
*P trend*		<.001	<.001	<0.01	<.001	<.001	<0.01

Values are average BP and 95% confidence interval.

Model 1: Adjusted for age and gender.

Model 2: Additionally adjusted for BMI, educational level, smoking, and alcohol consumption.

Model 3: Additionally adjusted for total energy, saturated fatty acids, carbohydrates, fiber, calcium, magnesium, potassium, and protein intake from other sources than the one under study, if applicable.

**Figure 1:**
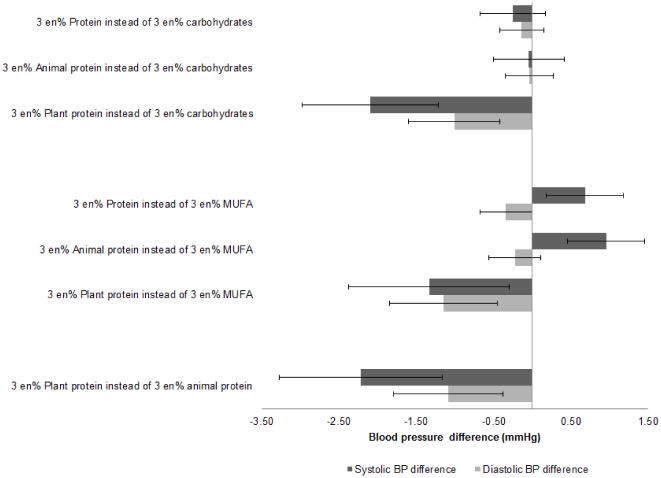
Fully adjusted systolic BP difference (mmHg) associated with replacement of 3 en% of carbohydrates or fat by total, plant or animal protein and by replacement of animal protein by plant protein.

Substitution analysis in which 3 energy% of carbohydrates or MUFA was substituted by total or animal protein did not show a difference in BP (**figure 1**). However, when 3 en% of carbohydrates was substituted by plant protein, BP was −2.1/−1.0 mmHg lower (p<0.01). Also substitution of 3 en% of mono-unsaturated fatty acids by plant protein resulted in a lower BP (−1.3/−1.2 mmHg, p<0.05)

With respect to protein from specific sources, systolic BP in the highest quintile of dairy protein intake was 1.6 mmHg higher than in the lowest quintile (p_trend_<0.01), which we did not observe for diastolic BP (**Table 3**). Intake of meat protein or grain protein was not associated with BP. With respect to plant protein from other sources than grain, systolic BP was +0.8 mmHg higher in the highest (median intake = 5.2 g/d) than in the lowest (1.4 g/d) tertile of potato protein. (p_trend_ = 0.01). For protein intake from vegetables (2.9 g/d in highest vs. 1.3 g/d in lowest tertile), fruits (2.0 vs. 0.4 g/d), and legumes (1.2 vs. 0.1 g/d) this difference in systolic BP was −0.9 mmHg (_ptrend_<0.01), +0.1 mmHg (p_trend_ = 0.50), and +0.8 mmHg (p_trend_<0.01), respectively.

**Table 3:** Fully adjusted systolic and diastolic BP in 20,820 untreated Dutch adults in quintiles of dairy, meat and grain protein intake.

	Median intake(g)	SBP	DBP
*Dairy protein*			
Q1	9	119.0 (118.4–119.7)	76.0 (75.5–76.4)
Q2	15	119.8 (119.4–120.3)	76.0 (75.7–76.3)
Q3	21	119.9 (119.5–120.3)	76.1 (75.8–76.4)
Q4	26	120.6 (120.2–121.1)	76.2 (75.9–76.5)
Q5	36	120.6 (119.9–121.3)	76.4 (75.9–76.9)
*ptrend*		<0.01	0.24
*Meat protein*			
Q1	9	119.5 (119.0–120.0)	75.8 (75.5–76.1)
Q2	16	120.3 (119.9–120.8)	76.2 (75.9–76.5)
Q3	21	120.4 (120.0–120.8)	76.7 (76.4–77.0)
Q4	25	120.2 (119.8–120.6)	76.0 (75.7–76.3)
Q5	32	119.5 (119.1–120.0)	76.0 (75.6–76.3)
*ptrend*		1.00	0.83
*Grain protein*			
Q1	9	119.9 (119.5–120.4)	76.3 (76.0–76.6)
Q2	13	120.5 (120.0–120.9)	76.4 (76.1–76.7)
Q3	15	119.7 (119.3–120.1)	76.0 (75.7–76.3)
Q4	18	120.2 (119.7–120.6)	76.3 (76.0–76.6)
Q5	22	119.7 (119.2–120.2)	75.7 (75.4–76.0)
*ptrend*		0.42	0.03

Values are average BP and 95% confidence interval, adjusted for age, gender, BMI, educational level, smoking, alcohol consumption, total energy, saturated fatty acids, carbohydrates, fiber, calcium, magnesium, potassium, and protein intake from other sources than the one under study, if applicable.

**Figure 2:**
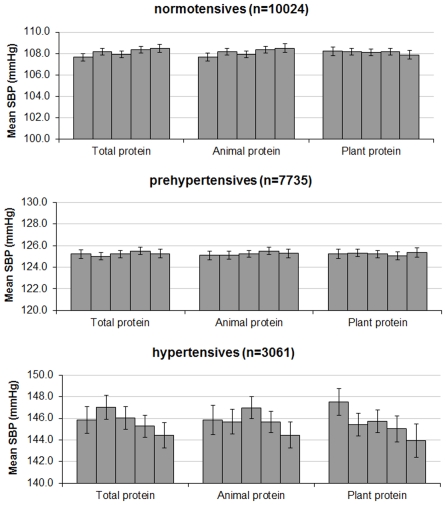
Systolic blood pressure in quintiles of protein intake, stratified by hypertension status. SBP = systolic blood pressure. p_interaction_ for total protein = 0.14, p_interaction_ for animal protein = 0.16, p_interaction_ for plant protein = <0.01. Values are average BP and 95% confidence interval, adjusted for age, gender, BMI, education, smoking, alcohol consumption, total energy, saturated fatty acids, carbohydrates, fiber, calcium, magnesium, potassium, and protein intake from other sources than the one under study, if applicable.

Age, gender, and BMI did not independently modify the associations between protein intake and BP (data not shown). The association between total protein intake and BP was not significantly modified by BP level (p_interaction_ = 0.14, **Figure 2**). With regard to protein types we observed no effect modification of BP level on the relation between animal protein and BP (p_interaction_ = 0.16), whereas plant protein was inversely associated with systolic BP in untreated hypertensives (−3.6 mmHg, p_trend_<0.01) but not in normotensives (−0.1 mmHg, p_trend_ = 0.39) and prehypertensives (+0.2 mmHg, p_trend_ = 0.97, p_interaction_<0.01).

## Discussion

In this cross-sectional study in 20,820 Dutch adults aged 20–65 years, total dietary protein and animal protein were not related to BP. High intake of plant protein was associated with lower BP, which was most pronounced in untreated hypertensive individuals. Protein from meat and grain were not related to BP, whereas dairy protein was directly associated with systolic, but not diastolic BP.

We conducted the current study among a large population of 20,820 Dutch adults. Protein intake is usually tightly regulated [23] and we consider it likely that protein intake measured in this study gives a good estimate of lifelong exposure. Nevertheless, due to the cross sectional design of the study it is possible that participants at increased cardiovascular risk, changed their diet upon medical advice. For this reason, we excluded individuals with diabetes, prevalent cardiovascular diseases, and clinically diagnosed hypertension (i.e. using antihypertensive medication). Because elevated BP is often asymptomatic we consider intentional dietary changes unlikely in participants that are not aware that they have a high BP. However, a total of 3999 participants (19%) reported that high blood pressure had ever been observed. Intakes of protein types of this group were not different from those in other participants (total protein: 15±2 en% for both groups; animal protein: 9.3±2.5 en% vs. 9.7±2.5 en%; plant protein 6±1 en% for both groups). Also intake of nutrients that are indicators of a healthy lifestyle were similar between the groups; fiber intake in the group with a history of high BP was 24±7 g/d versus 25±8 g/d in the other group, and potassium intake was 3675±961 mg/d versus 3748±1019 mg/d. Therefore, we do not expect that reverse causality has influenced our findings.

Extensive data collection in this large population based cohort allowed adjustment for many potential confounders. Nevertheless, physical activity, which is an important BP determinant, was not assessed until 1994 and data were available for only 77% of our cohort. In this subgroup physical activity appeared not to confound the association between dietary protein and BP. We therefore consider it unlikely that lack of adjustment for physical activity has affected our findings.

Protein intake in the present study was assessed using a self-administered semi-quantitative FFQ. Validation against 24-hour dietary recalls and 24-hour urine samples showed good correlations for total dietary protein (all correlation coefficients >0.55), indicating that participants could be adequately ranked according to their protein intake [21]. However, the FFQ was not validated for protein types. Although correlations with 24-h recalls were good for milk and bread, as surrogate markers for protein from dairy and grain, correlations for meat, as surrogate marker for meat protein, were lower, especially in men (r = 0.39) [19]. Misclassification of participants, especially for meat protein, may have led to attenuated associations with BP, and these findings should therefore be interpreted with caution.

The lack of significant association between total protein and BP in our study is in agreement with previous observational studies showing inconclusive results [6]. Results of trials, however, suggest that protein may have a small beneficial effect on BP [5], [6], [24], [25]. Most of these trials had a carbohydrate-rich control diet. The fully controlled Omniheart trial in 164 US adults additionally compared a protein rich diet with an isocaloric diet that was rich in mono-unsaturated fat [5]. BP was similar during these diets, and the authors therefore argued that reduced carbohydrate rather than increased protein intake lowers BP. We could not confirm this hypothesis with our substitution analysis that yielded no association of dietary protein with BP, irrespective of whether protein was exchanged with carbohydrates or monounsaturated fat. This discrepancy may be explained by contrast in protein intake, which was only 4 en% between extreme quintiles in the present study whereas it was 10 en% in Omniheart. Moreover, BP in our cohort was low (120/76 mmHg) compared to that of (pre)hypertensive trial participants.

In our analysis plant protein was inversely associated with BP, whereas we observed no association for animal protein. In OmniHeart [5], BP reductions may have been due to extra intake of plant protein, which accounted for two thirds of the difference in protein intake between the diets. A differential effect of dietary plant and animal protein on BP might be explained by differences in amino acid composition. In the INTERMAP study in 4,680 adults, individuals with a high intake of plant protein also had a relatively high intake of glutamic acid [26]. With a 2 SD higher intake of glutamic acid (4.7% of total protein) the authors observed 1.5 mmHg lower systolic and 1.0 mmHg lower diastolic BP levels. On the other hand, although we adjusted our estimates for many potential confounders including potassium and fiber as healthy diet indicators, we cannot exclude the possibility that unmeasured beneficial nutrients that are closely correlated to plant protein (e.g. polyphenols) or healthy lifestyle in general have contributed to the observed associations between plant protein and BP.

The inverse association of plant protein with BP could not be explained by grain protein, which comprised 48% of plant protein intake. Therefore we performed post-hoc analysis to explore whether other sources of plant protein could explain the observed inverse association for plant protein. This was not the case for protein intake from potatoes, legumes, and fruits, which was either directly or not associated with BP. However, intake of vegetable protein, which contributed 7% to plant protein intake in our population, showed a small inverse relationship with BP and could possibly (partly) explain a beneficial association of plant protein with BP. On the other hand, a high vegetable protein intake may also be a marker for a healthy diet and lifestyle, which may have contributed to the observed inverse associations.

With respect to protein from animal sources, meat protein (40% of animal protein intake) was not associated with BP. This is in line with results from previous analysis in 2241 older Dutch adults of the Rotterdam cohort, where intake of meat protein was not related to hypertension risk [12]. Moreover, protein from several meat sources did not affect BP compared to plant protein or non-meat protein in a randomized controlled trial among 64 hospital staff members and a randomized controlled cross over trial among 35 men respectively [27], [28]. For dairy protein (42% of animal protein intake) we found a direct association with systolic, but not with diastolic BP. In the Rotterdam cohort dairy protein was not associated with incident hypertension [12]. Also, in a fully controlled weight loss trial including 65 adults, a diet containing 15 en% milk protein did not affect BP compared to a diet in which the milk protein was exchanged for fat [29]. Moreover, in a double-blind randomized cross-over trial including 352 (pre)hypertensive participants milk protein supplementation (40 g/d) resulted in a BP reduction of −2.3 mmHg compared to carbohydrate supplementation [15]. Therefore, the direct association between dairy protein and systolic BP that we observed in the current study may well be a chance finding.

Our results suggest that untreated hypertensive individuals could be more sensitive to a beneficial effect of plant protein than normotensive or prehypertensive individuals. This is in line with findings from the OmniHeart study [5], in which larger BP reductions were found for increased protein intake (largely from plant sources) in untreated hypertensives than in prehypertensives. Because over 30% of the global adult population is estimated to be hypertensive, this finding could have important public health implications and warrants further investigation.

In conclusion, intake of total protein and animal protein was not associated with BP in this general Dutch population not using antihypertensive medication. Our results suggest that plant protein may lower population BP level by ∼2 mmHg, especially in those with elevated BP levels. This may have important public health implications because a downward shift in population BP by 2 mmHg may reduce cardiovascular mortality by ∼5% [3]. However, due to the cross-sectional design a definitive conclusion on causality cannot be drawn. Moreover, we cannot exclude that high plant protein is a marker for a healthy lifestyle in general. Therefore, confirmation from randomized controlled trials is warranted.
